# A Spectral Detection Method Based on Integrated and Partition Modeling for Trace Copper in High-Concentration Zinc Solution

**DOI:** 10.3390/molecules29174006

**Published:** 2024-08-24

**Authors:** Fengbo Zhou, Bo Wu, Jianhua Zhou

**Affiliations:** Hunan Province Key Laboratory of Southwest, Hunan Academician Workstation, School of Information Science and Engineering, Shaoyang University, Shaoyang 422000, China; bwu4580@126.com (B.W.); fb.john@hnsyu.edu.cn (J.Z.)

**Keywords:** zinc hydrometallurgy, continuous wavelet transform, interval partition modeling, integrated modeling, ultraviolet visible spectroscopy

## Abstract

In zinc smelting solution, because the concentration of zinc is too high, the spectral signals of trace copper are masked by the spectral signals of zinc, and their spectral signals overlap, which makes it difficult to detect the concentration of trace copper. To solve this problem, a spectrophotometric method based on integrated and partition modeling is proposed. Firstly, the derivative spectra based on continuous wavelet transform are used to preprocess the spectral signal and highlight the spectral peak of copper. Then, the interval partition modeling is used to select the optimal characteristic interval of copper according to the root mean square error of prediction, and the wavelength points of the absorbance matrix are selected by correlation-coefficient threshold to improve the sensitivity and linearity of copper ions. Finally, the partial least squares integrated modeling based on the Adaboost algorithm is established by using the selected wavelength to realize the concentration detection of trace copper in the zinc liquid. Comparing the proposed method with existing regression methods, the results showed that this method can not only reduce the complexity of wavelength screening, but can also ensure the stability of detection performance. The predicted root mean square error of copper was 0.0307, the correlation coefficient was 0.9978, and the average relative error of prediction was 3.14%, which effectively realized the detection of trace copper under the background of high-concentration zinc liquid.

## 1. Introduction

The concentration of trace copper (Cu) in zinc (Zn) liquid is an important technical index of the zinc purification process in hydrometallurgy. A proper amount of copper ion can be used as an activator for cobalt removal, but its high concentration will reduce the electrolytic efficiency, so accurate detection of copper concentration is the premise and foundation for the stability of the electrolytic process [1,2,3,4,5]. Ultraviolet–visible spectrophotometry (UV–vis) has the advantages of high accuracy and a simple analysis method, and has been widely used in the detection of low-concentration polymetallic ions in solution [6,7,8]. However, in the industrial purification process of zinc hydrometallurgy, the concentration ratio of matrix zinc to copper ranges from 2500 to 6000, and there are the following problems in the detection process: First, because of the similar chemical characteristics between ions, the detection signals often overlap. Second, under the background of the high-concentration zinc solution, the concentration of zinc ions is nonlinear far beyond the linear range. Third, the high concentration of the zinc solution makes the absorbance no longer meet the superposition in the whole band. Fourth, the high concentration of the zinc solution makes the linearity of trace copper poor. The traditional modeling method uses all wavelength variables for partial least squares (PLS) modeling, and these wavelength points are not all useful information, even if there is a lot of noise information and interference information [9,10,11,12]. In order to solve this problem, it is necessary to extract the characteristics of copper spectral curves and select effective wavelength points for modeling.

With the development of molecular-spectral-analysis technology, the analysis and correction methods are constantly being developed and improved. The commonly used linear regression methods include principal component analysis (PCR) and partial least squares (PLS), and the nonlinear correction methods include artificial neural networks (ANNs) and support vector regression (SVR) [13,14,15,16,17,18,19,20]. The interference and overlap of the spectral signals of metal ions in zinc liquid are serious, and the traditional analysis and correction methods are based on the full spectral band and contain a lot of noise and redundant information, which makes it difficult to meet the requirements of detection accuracy [21,22,23,24,25]. In view of this limitation, scholars at home and abroad have conducted relevant research on the screening methods of effective wavelength variables. The commonly used methods for selecting characteristic variables mainly include: interval partial least squares (IPLS), joint interval partial least squares (SIPLS), the Monte Carlo elimination method without information variables (MC-UVE), and the competitive adaptive weighting algorithm (CARS) [26,27,28]. Silva et al. [29] used the IPLS method to determine the composition of LDPE/HDPE blends, and evaluated the influence of the subinterval number (10–50) and interval combination (2–4) on the prediction performance, which effectively improved the detection performance. Zhu et al. [30] applied the SIPLS method to detect cadmium content in rice by near infrared spectroscopy. The results showed that the SIPLS method could not only decrease the complexity of the model, but it also improved the prediction precision. Hao et al. [31] used the MC-UVE method to analyze the hardness and surface color of pears by near infrared spectroscopy, which reduced the modeling variables from the initial 1451 to 220, and significantly improved the operation speed and detection accuracy. Liu et al. [32] used the CARS method to verify the data set of a feed-protein fermentation process. The experimental results showed that the CARS method could improve the accuracy and stability of near infrared characteristic wavelength selection.

The above methods can select the wavelength well in some cases, but this is not universal, especially in the background of a high-concentration matrix. IPLS and SIPLS both model the interval band, so they cannot analyze every wavelength point, and may select the wavelength point that contributes little to the model just because the wavelength point is in the interval. Although MC-UVE and CARS can analyze each wavelength point, the sampling process of MC-UVE is random, and the calculation of the stability index of induced variables is inaccurate. In addition, the purpose of MC-UVE is to eliminate noise wavelength points and non-noise points with a low contribution to modeling, which has great limitations. The CARS method has a good index at the peak of the matrix ion, but it is difficult to choose the wavelength point of matrix zinc information, which leads to the inaccuracy of the copper model [33,34,35,36]. Therefore, it is very difficult to detect the concentration of polymetallic impurity ions in a high-concentration zinc solution.

Aiming at the characteristics of interference, masking, serious overlap, and strong nonlinearity of spectral signals of zinc solution, our team’s previous research results provide a good reference [37,38]. However, the fluctuation in the zinc matrix, random noise of detection instruments, mutual interference and inhibition of polymetallic ions, and interference of the reagent background, all affect the spectral signal of copper, thus affecting the accuracy and repeatability of a single sample, and it is difficult to achieve robust detection of all samples. Therefore, this paper proposes a spectrophotometric method based on integrated and partition modeling to establish the model between spectral signals of a mixed solution and trace copper concentration. Firstly, the derivative method of continuous wavelet transform is used to highlight the masked copper characteristic information. Then, the wavelength is preliminarily selected by the interval partition method, the prediction error of the model under each interval wavelength is calculated, and the variable set with high correlation among wavelength variables is selected as the modeling wavelength by the correlation-coefficient threshold. Finally, the PLS integrated-modeling method is used to establish the regression model between copper concentration and absorbance, which improves the accuracy and robustness of the PLS model and realizes the detection of trace copper in a high-concentration zinc solution.

## 2. Theory

### 2.1. Interval Partition Wavelength Selection

In order to solve the problem that the spectral signals of copper and zinc overlap under the background of a high-concentration zinc solution, and the absorbance and concentration of the solution are strongly nonlinear in some wavelength bands, it is impossible to detect the concentration of trace copper through all-band information. Therefore, an interval-partition-modeling method is proposed. This method uses the interval correlation coefficient to screen the wavelength points of spectral signals, and then establishes a PLS model to detect copper concentration according to the screened wavelengths. The whole spectra are divided into several sub-intervals, and the predicted root mean square error (RMSEP) and correlation coefficient (R2) of the whole spectral model and each sub-interval model can be obtained by PLS modeling, and the sub-intervals with small RMSEP are selected for modeling. Abs represents the absorbance matrix, and the RMSEP and R2 are set as follows:

(1) $$\mathrm{Abs}=\left[\begin{array}{cccc} y_{11} & y_{12} & \cdots & y_{1n}\\ y_{21} & y_{22} & \cdots & y_{2n}\\ \vdots & \vdots & \vdots & \vdots \\ y_{m1} & y_{m2} & \cdots & y_{mn} \end{array}\right]$$

(2) $$RMSEP=\sqrt{\frac{\sum_{i=1}^{n}{\left({\hat{y}}_{i}-y_{i}\right)}^{2}}{n}}$$

(3) $$R^{2}=\frac{\sum_{i=1}^{n}{\left({\hat{y}}_{i}-\bar{y}\right)}^{2}}{\sum_{i=1}^{n}{\left(y_{i}-\bar{y}\right)}^{2}}$$

where *m* is the number of samples and *n* is the number of wavelengths. $y_{i}$ is the actual value of the i-th wavelength, ${\hat{y}}_{i}$ is the predicted value, and $\bar{y}$ is the average value. r represents correlation rate, and is given by

(4) $$r_{\mathrm{ij}}=\frac{\sum_{k=1}^{n}\left(y_{\mathrm{ki}}-{\bar{y}}_{i})(y_{\mathrm{kj}}-{\bar{y}}_{j}\right)}{\sqrt{\sum_{k=1}^{n}{\left(y_{\mathrm{ki}}-{\bar{y}}_{i}\right)}^{2}\sum_{k=1}^{n}{\left(y_{\mathrm{kj}}-{\bar{y}}_{j}\right)}^{2}}}$$

Then, the correlation matrix $R_{r}$ can be written as follows:

(5) $$R_{r}=\left[\begin{array}{cccc} r_{11} & r_{12} & \cdots & r_{1n}\\ r_{21} & r_{22} & \cdots & r_{2n}\\ \vdots & \vdots & \vdots & \vdots \\ r_{n1} & r_{n2} & \cdots & r_{nn} \end{array}\right]$$

**Figure 1:**
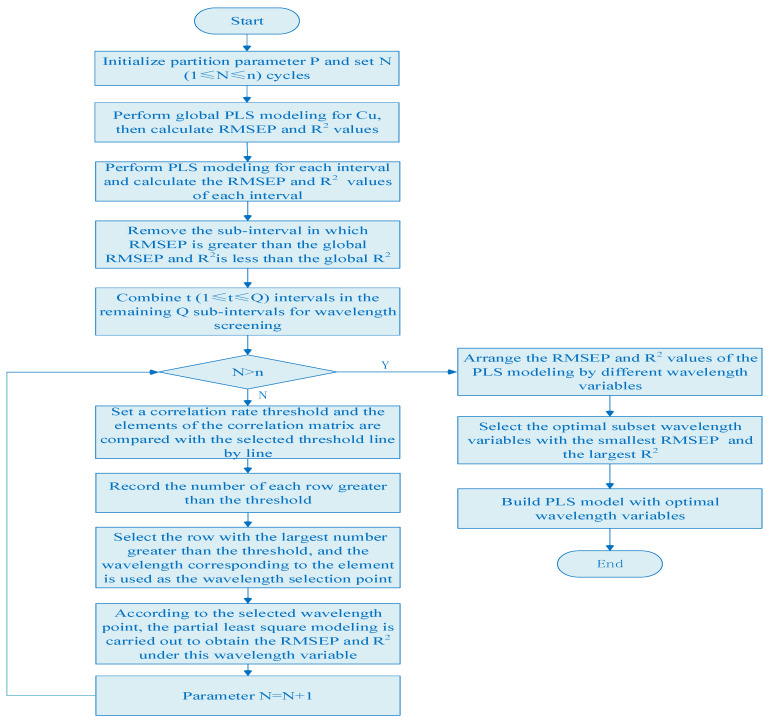
The flow chart of interval partition wavelength selection.

The proposed algorithm first uses interval partition modeling to roughly select wavelengths, and then uses the correlation-coefficient threshold to finely select wavelength points. The basic steps of the algorithm are:

(1) The global PLS model of copper is established in the full spectral range, and the RMSEP and R^2^ values of copper are calculated according to Formulas (2) and (3) as thresholds for interval screening.
(2) The whole spectra is divided into P subintervals with equal width, the local PLS model of copper is established in each subinterval, and the RMSEP and R^2^ values of copper in P subintervals are calculated.
(3) The RMSEP and R^2^ values of the full spectral model and each local model are compared, the wavelength interval in which RMSEP is greater than the global RMSEP and R^2^ is less than the global R^2^ is removed, and the remaining Q subintervals with smaller RMSEP and larger R^2^ values are taken out.
(4) N (1≤N≤n) cycles are set and a correlation-rate threshold is set for each cycle. The elements of the correlation matrix $R_{r}$ are compared with the selected threshold line by line.
(5) The number of each row greater than the threshold is recorded, and the row with the largest number greater than the threshold is selected. The wavelength corresponding to the element in this row that is greater than the correlation-rate threshold is used as the wavelength selection point.
(6) According to the selected wavelength point, partial least squares modeling is carried out to obtain the RMSEP and R^2^ under this wavelength variable, and the next cycle is carried out.
(7) When the cycle is over, the modes established by different wavelength variables are compared, and the subset with the smallest RMSEP value and the largest R^2^ is the optimal subset. The variable filtering process is then complete. The algorithm flow chart is shown in Figure 1.

### 2.2. PLS Integrated Modeling Based on Adaboost Algorithm

Factors such as the fluctuation in matrix zinc, random noise of testing instruments, mutual interference and inhibition of multi-metal ions, and reagent background interference all affect the spectral signal of copper, thus affecting the accuracy and repeatability of a single sample, and it is difficult to achieve robust detection of all samples. In order to solve the above problems, this paper proposes a PLS integrated-modeling method based on the Adaboost algorithm. The advantage of integrated modeling is that by using the information of different variables in the training set randomly many times to model, the weight of variables is changed, and the dependence of the final strong model on unstable variables is reduced, which improves the stability of the model.

Adaboost is a branch of Boosting, and it is an iterative algorithm. Its core idea is to establish multiple weak models through different variables, then train these weak models with the same training set to get a series of basic models and their corresponding weights, and, finally, integrate these basic models according to certain rules to construct a strong model. In this paper, based on the characteristics of UV–vis spectra, the integrated-modeling strategy is applied to the analysis of trace copper in a zinc hydrometallurgy solution. With partial least squares (PLS) as the basic learning algorithm, multiple member models are established, and the Adaboost algorithm is used to predict the concentration of trace copper in the zinc solution at the same time. The following are the specific implementation steps:

(1) The weight distribution of training data is initialized:

(6) $$D_{l}=\{w_{ll},\cdot \cdot \cdot w_{li},\cdot \cdot \cdot ,w_{lN}\},w_{li}=1/N,i=1,2,\cdot \cdot \cdot ,N$$

(2) The sample weights are trained iteratively, and the threshold of iteration times is MT. The threshold of iteration times used in this paper is 30, and m represents the m-th iteration.
(3) Several PLS weak models are generated by random sampling, and the weak model with the lowest error rate is selected as the m-th basic model to predict the verification samples, and the error rate is calculated:

(7) $$\varepsilon_{m}=\sum_{i=1}^{N}w_{mi}I(e_{2}(i))$$

(8) $$I(x)=\left\{\begin{array}{l} 10\times (x-10\%)\\ 0 \end{array}\right.\begin{array}{l} x>10\%\\ x\leq 10\% \end{array}$$

(4) The weight of the m-th basic model Hm(x) in the final model is calculated. The proportion of the basic model is negatively correlated with the error rate. The higher the prediction error rate of this model, the lower the proportion of this model in the final model:

(9) $$\alpha_{m}=\frac{1}{2}\ln(\frac{1-\varepsilon_{m}}{\varepsilon_{m}})sign(\frac{1}{2}\ln(\frac{1-\varepsilon_{m}}{\varepsilon_{m}}))$$

(5) The weight distribution of the training data set is updated:

(10) $$D_{m+1}=\{w_{m+1,l},\cdot \cdot \cdot w_{m+1,i},\cdot \cdot \cdot ,w_{m+1,N}\},i=1,2,\cdot \cdot \cdot ,N$$

(11) $$w_{m+1,i}=\frac{w_{mi}\exp(-\alpha_{m}y_{i}\hat{y_{i}})}{Z_{m}}$$

(12) $$Z_{m}=2\sqrt{\varepsilon_{m}(1-\varepsilon_{m})}$$

where $Z_{m}$ represents the normalization factor, $y_{i}$ represent the calibration value of the i-th sample, and ${\hat{y}}_{i}$ represent the predicted value.

(6) The number of iterations is then checked to ensure that it does not exceed the iteration threshold. If the number of iterations exceeds MT, the basic model is combined as shown in Formula (13) to obtain the final classifier:

(13) $$f(x)=sign\left(\sum_{m=1}^{M_{r}}\alpha_{m}H_{m}(x)\right)$$

## 3. Results and Discussion

### 3.1. Spectral Characteristics

**Figure 2:**
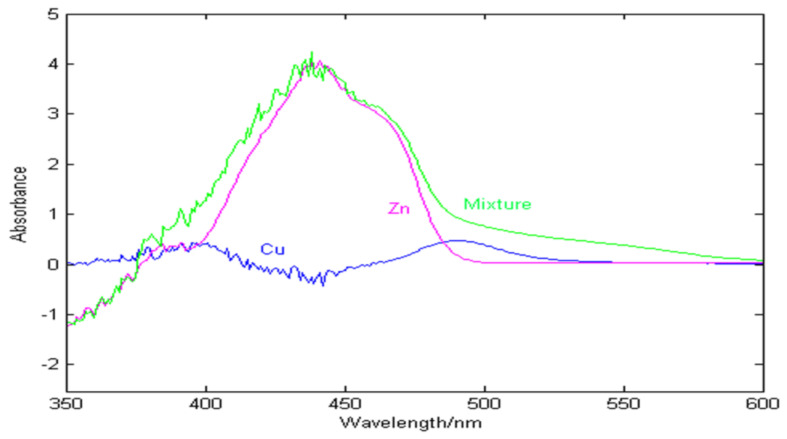
The absorption spectra of zinc, copper, and their mixtures.

In the purification process of zinc hydrometallurgy, zinc sulfate solution usually contains a lot of zinc and impurity metal ions, and the concentration of zinc and copper presents a high concentration ratio. Figure 2 shows the absorption spectra of zinc (20 g/L), copper (1.2 mg/L), and their mixtures in the wavelength range of 350–600 nm. As can be seen from the Figure 2, the concentration of zinc is much greater than that of copper, and almost close to the spectral signal of their mixture, which leads to the low sensitivity of copper and is easily masked by noise signals.

**Figure 3:**
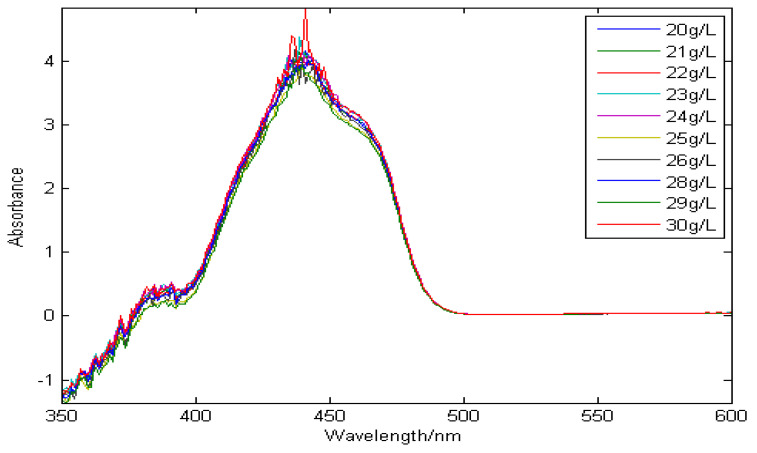
The absorption spectra of zinc.

Moreover, the spectra of copper and zinc overlap seriously, which makes the spectral resolution of copper ions low and the effective band narrow. Therefore, the problems of low sensitivity and narrow effective band seriously affect the detection accuracy of copper ions in zinc sulfate solution. Figure 3 shows a set of absorption spectral signals of zinc in the wavelength range of 350–600 nm, in which the concentration range of zinc is 20–30 g/L. As can be seen from the Figure 3, with the increase in zinc concentration, the absorption spectra of zinc are almost unchanged. The reason for this is that the concentration of zinc ion in the solution was supersaturated, which led to the relationship between absorbance and concentration not satisfying the Lambert–Beer law. Therefore, when measuring the concentration of copper in zinc sulfate solution, a 20 g/L high-zinc solution can be used as reference solution to eliminate the interference of matrix zinc as much as possible.

### 3.2. Derivative Spectrometry

**Figure 4:**
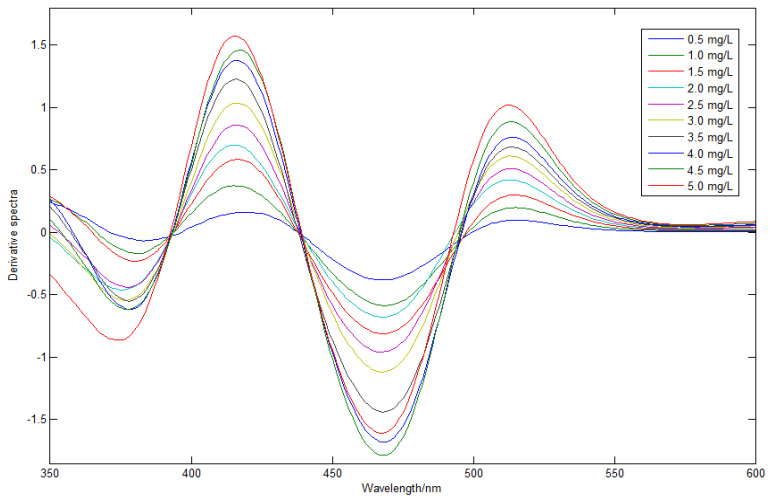
Derivative spectral signal based on continuous wavelet transform.

In order to solve the problem that the copper spectra were completely covered by zinc spectra, derivative spectroscopy was used to improve the spectral resolution of copper. The continuous wavelet transform was used to approximate the derivative, where the wavelet function was the Haar function and the decomposition level was 20. With 20 g/L zinc solution as reference, the derivative spectral signals of copper, based on continuous wavelet transform, are shown in Figure 4, where the concentration range of copper was 0.5–5 mg/L. After derivative processing, the characteristics of the copper signal began to show, and the copper peak was reconstructed. The characteristic peaks of the copper derivative spectra were at wavelengths of 418 nm and 515 nm, which significantly improved the sensitivity of the copper. In order to evaluate the linearity of the copper derivative spectra, several wavelength points were randomly selected for linear fitting of absorbance and concentration. The calculation results of the linear regression equation and the correlation coefficient (R^2^) are shown in Table 1.

The correlation coefficient (R^2^) was used to characterize the linear correlation between the copper derivative spectra and its concentration. The larger the R^2^ value, the better the copper linearity. As can be seen from Table 1, copper ions had good linearity at wavelengths of 415 nm, 509 nm, and 540 nm, but had no linearity at wavelengths of 497 nm and 368 nm. Due to the influence of matrix-zinc interference and instrument noise, the data show that copper ions had no linearity at some wavelengths, but had good linearity at some wavelengths. Therefore, in order to improve the detection accuracy of copper, it is necessary to optimize the wavelength variable in PLS modeling.

### 3.3. Application of Interval Partition Method

**Figure 5:**
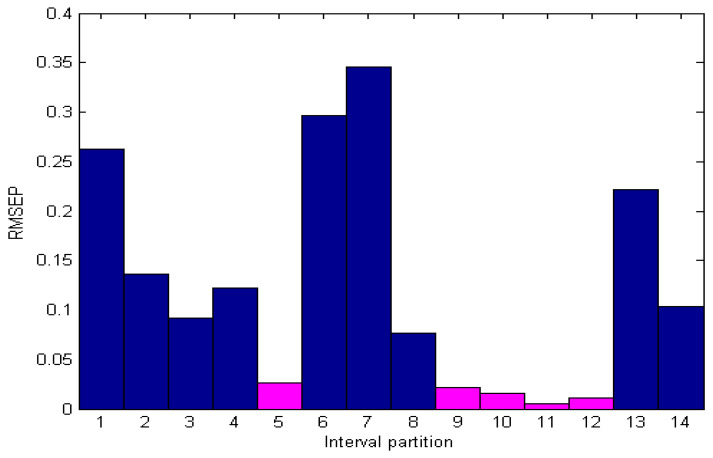
The predicted root mean square error of interval partition.

According to the interval partition wavelength selection method described in Section 2.1, 251 wavelength variations (350–600 nm, resolution 1 nm) of the derivative spectra of copper were selected. Starting from the wavelength point of 350 nm, every 18 wavelength points was an interval, and PLS modeling was carried out for 14 intervals, and the predicted root mean square error (RMSEP) of each wavelength interval was obtained, as shown in Figure 5. As can be seen from Figure 5, the RMSEP between regions 5, 9, 10, 11, and 12 was relatively small, and its wavelength bands were 423–440 nm and 495–566 nm, so the next screening was carried out with the wavelength points of this wavelength band as variables.

**Figure 6:**
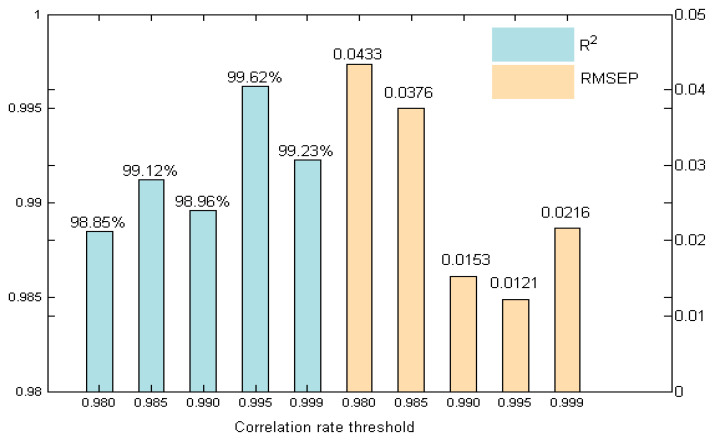
The RMSEP and R^2^ under different correlation-rate thresholds.

In the selected ranges of 423–440 nm and 495–566 nm, the change in zinc concentration will still change the absorbance of the mixed solution, which is unpredictable because its absorbance and concentration are nonlinear. If the absorbance coefficient of copper at a certain wavelength is small, the difference in absorbance produced by different concentrations of zinc is similar to that of copper, and the change in zinc concentration will seriously affect the measurement of trace copper. Therefore, we chose the correlation-rate threshold to obtain the characteristic wavelengths, so that the absorbance change caused by the change in zinc concentration had little influence on the absorbance of copper. We set five cycles, and selected 0.98, 0.985, 0.99, 0.995, and 0.999 as correlation-rate thresholds. We compared each row of the correlation matrix $R_{r}$ in Equation (3) with the selected threshold, recorded the number of each row greater than the threshold, selected the row with the largest number greater than the threshold, and took the wavelength corresponding to this row as the characteristic wavelength point. Each correlation-coefficient threshold corresponded to a set of modeled wavelength points. Based on partial least squares modeling with each set of modeling wavelength points, five different RMSEP and R^2^ were obtained, as shown in Figure 6.

As can be seen from Figure 6, by comparing RMSEP and R^2^ with different correlation-rate thresholds, it was found that when the correlation-rate threshold was 0.995, the predicted root mean square error (RMSEP) was the smallest, with a value of 0.0121. The correlation coefficient was the largest, with a value of 99.62%. At this time, the corresponding wavelength point was the optimal subset of the model, with a total of 47 variables, and the selected wavelength range was 510–556 nm.

### 3.4. Integrated Modeling Based on Adaboost Algorithm

Due to the simultaneous action of many factors in the actual measurement process, some samples will be pulled up due to random noise. These problems will lead to the high absorbance of some samples at the characteristic wavelength points due to excessive noise, which makes the established absorbance–concentration regression model not ideal in accuracy and stability when detecting these samples which are high due to random noise, and it is difficult to realize the robust detection of all samples. In order to solve the above problems, this paper further improves PLS modeling method and proposes a PLS integrated-modeling method based on the Adaboost algorithm. This algorithm takes PLS as the basic sub-model, and reduces the weight of weak classifiers with a high classification error rate and increases the weight of weak classifiers with a low classification error rate through the weighted combination of multiple basic models, thus improving the sample detection accuracy, enhancing the model stability, and realizing the stable detection of trace copper in a high-concentration zinc solution.

**Figure 7:**
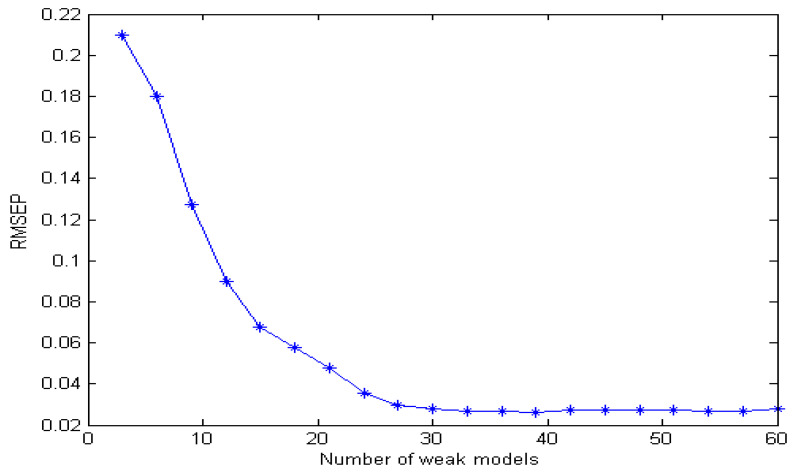
Relationship between estimation error and the number of weak models.

The more important factor of integrated modeling is the number of weak models. If the number of weak models is too large, it will increase the complexity of calculation and affect the real-time detection. When the number of weak models is too small, the random combination can not fully cover all the characteristic wavelength information, thus affecting the accuracy of the results. Figure 7 is an estimation error diagram using different models. It can be seen from the figure that with the increase in the number of weak models, the estimation error of the model decreases gradually. When the number of weak models exceeds 30, the model tends to be gradually become stable, and the estimation error is basically unchanged with the increase in the number of weak models. Therefore, this paper sets the number of weak models at 30.

### 3.5. Performance Comparison of Different Algorithms

**Figure 8:**
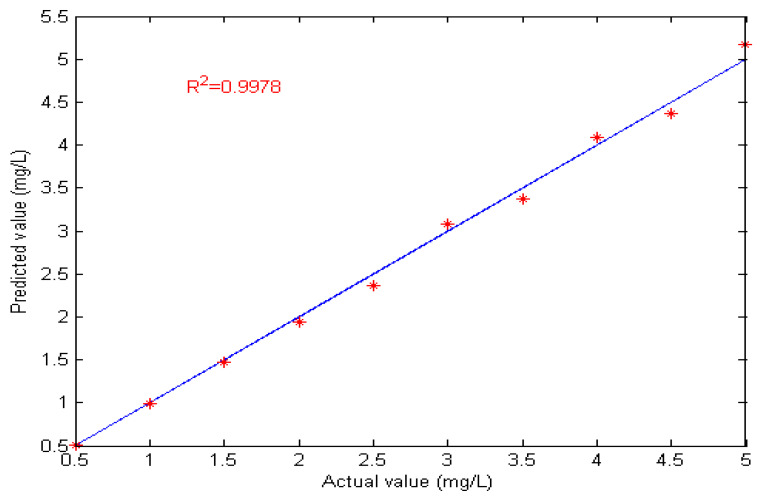
Predicted value and actual value of copper.

The proposed spectrophotometric method based on integrated and partition modeling (IPM) was used to detect copper concentration under the background of high-concentration zinc liquid, as shown in Table 2. It can be seen that the maximum relative error of copper was 5.68%, the average relative error was 3.02%, the limit of detection (LOD) was 0.14 mg/L, and the RMSEP was 0.031. In order to further verify the stability of the IPM method, 30 repeatability tests were performed on the copper experimental data, and the full-band partial least squares method (FBPLS), Monte Carlo uninformative variable elimination method (MC_UVE), and competitive adaptive weighting method (CARS) were used to detect copper concentration. The performance comparison results are shown in Table 3. The predicted and actual concentration of Cu by the IPM method is shown in Figure 8.

As can be seen from Table 3, the wavelength range of the four methods was 350–600 nm. According to the characteristic wavelength selection criteria of each method, the wavelength numbers of the four methods were 251, 65, 82, and 47, respectively, because there was a lot of redundancy, interference information, and induced analysis in the full-band modeling. The accuracy of the FBPLS model was low, and its real-time performance was poor because of many modeling variables. When CARS and MC_UVE selected variables, many wavelength points were in the zinc-sensitive area, which made the model accuracy worse. The variables screened by the proposed IPM method had great correlation in the sensitive area of copper, so the wavelength points containing copper information could be better selected, with less variation, better real-time performance, lower predicted root mean square error (RMSEP), and a higher correlation coefficient (R^2^). The maximum relative error of Cu was 6.22%, the average relative error was 3.14%, and the RMSEP was 0.0307. The average relative error was less than 5%, which meets the industrial detection index. As can be seen from Figure 8, the predicted value was basically consistent with the actual value. The RMSEP of copper was 0.0307, and the R^2^ of copper was 0.9978. The results show that the proposed IPM method can effectively detect copper in zinc liquid.

## 4. Experimental

### 4.1. Apparatus and Reagents

A T9 dual-beam ultraviolet-visible spectrophotometer (Purkinje General Instrument Ltd., Beijing, China) was selected as the instrument. The spectrometer adopts a double-beam optical structure, which can counteract the influence of stray light, electrical noise, and light-source fluctuation, which is beneficial to improving the accuracy and stability of instrument detection. A nitroso R salt solution was used as chromogenic agent with a mass fraction of 0.4%. Acetic acid–sodium acetate was used as buffer solution, and its pH was 5.5. Hexadecyl trimethyl ammonium bromide (0.01 mol/L) was used as the stabilizer solution. The mass concentration of the Zn standard solution was 180 g/L. The mass concentration of the Cu standard solution was 50 mg/L. All chemicals were analytical reagents (AR) without further purification.

### 4.2. Procedures

Zinc in the mixed solution was a matrix ion with high mass concentration, and copper was a trace ion to be detected. According to the experimental requirements, a certain amount of the zinc and copper standard solution was transferred into a 25 mL volumetric flask. Then, add 2 mL nitroso R salt solution, 5 mL acetic acid-sodium acetate buffer, and 3 mL hexadecyl trimethyl ammonium bromide were added in turn, and the mixture was diluted with deionized water to a constant volume. The concentration range of zinc was 20–30 g/L. The concentration range of copper was 0.5–5.0 mg/L. Taking the reagent blank (excluding zinc and copper) as reference, the absorbance of each wavelength point was measured at intervals of 1 nm in the wavelength range of 350–600 nm. Then, 40 groups of absorption spectral signals were measured in the experiment, of which 30 groups were used as calibration sets and 10 groups as prediction sets.

## 5. Conclusions

In high-concentration zinc solution, because the spectral signal of trace copper is masked and interferes seriously, a spectrophotometric method based on integrated and partition modeling is proposed to solve the problem that copper ions are difficult to detect. Compared with the traditional full-band PLS modeling method, the proposed method effectively eliminates the wavelength points with large noise and serious spectral line overlap. Compared with MC-UVE or CARS modeling method, this proposed method improves the stability and applicability of wavelength variable screening by setting the threshold of correlation coefficient; it selects the wavelength variable with high correlation with copper, and then uses a PLS integrated-modeling method to build the model. The experimental results show that the proposed method achieves good results, and the precision of the model is obviously improved, which solves the problem of trace copper concentration detection under the background interference of high-concentration zinc liquid. The proposed method can also be applied to fluorescence spectroscopy, laser-induced breakdown spectroscopy, infrared absorption spectroscopy, and other fields, combining with spectral characteristics to analyze data and establish a quantitative regression analysis model to solve the problems of overlap and interference.

## Figures and Tables

**Table 1:** Linear index of copper ions at different wavelength points.

Wavelength/nm	Linear Regression Equation	Correlation Coefficient (R^2^)
368	Y = 0.1150x − 0.1414	0.6667
415	Y = 0.0650x + 0.3081	0.9906
444	y = −0.0607x − 0.0528	0.8257
497	y = −0.0879x + 0.0283	0.3791
509	y = −0.0147x + 0.1807	0.9891
540	y = 0.0076x + 0.0698	0.9932

**Table 2:** The predicted concentrations of Cu by IPM method.

No.	Actual Value (mg/L)		Predicted Value (mg/L)	Relative Error (%)
	Zn	Cu	Cu	Cu
1	2.1 × 10^4^	2.00	1.938	3.10
2	2.2 × 10^4^	4.00	4.093	2.33
3	2.3 × 10^4^	0.50	0.513	2.60
4	2.4 × 10^4^	2.50	2.358	5.68
5	2.5 × 10^4^	4.50	4.373	2.82
6	2.6 × 10^4^	1.00	0.984	1.60
7	2.7 × 10^4^	3.00	3.086	2.87
8	2.8 × 10^4^	5.00	5.171	3.42
9	2.9 × 10^4^	1.50	1.467	2.20
10	3.0 × 10^4^	3.50	3.376	3.54
Average relative error (%)				3.02
LOD (mg/L)				0.14
RMSEP				0.031

**Table 3:** The modeling comparison of four feature extraction methods.

Detect Ion	Evaluation Index	FBPLS	CARS	MC_UVE	IPM
Cu	Wavelength number	251	65	82	47
	Maximum relative error/%	74.31	21.35	11.29	6.22
	Average relative error/%	19.52	9.27	7.73	3.14
	R^2^	0.8833	0.9674	0.9916	0.9978
	RMSEP	0.2782	0.1345	0.0833	0.0307

## Data Availability

The data presented in this study are available on request from the corresponding author.
